# Cognitive strategies to regulate emotions—current evidence and future directions

**DOI:** 10.3389/fpsyg.2013.01019

**Published:** 2014-01-10

**Authors:** Natali Moyal, Avishai Henik, Gideon E. Anholt

**Affiliations:** Department of Psychology and the Zlotowski Center for Neuroscience, Ben-Gurion University of the NegevBeer-Sheva, Israel

**Keywords:** emotion regulation, reappraisal, distraction, labeling, cognitive strategies

Emotions are important and basic in human experience, and are comprised of different components, such as subjective feelings, cognitive appraisal, physiological response and action tendencies (Kleinginna and Kleinginna, 1981). Emotions become dysfunctional when they interfere with one's ability to behave adaptively, and therefore successful emotion regulation (ER), when necessary, is crucial for psychological health. Difficulties in adaptive ER are related to different psychopathologies such as depression and anxiety disorders [for a review see Aldao et al. (2010)]. A well-known model that describes the process of ER was suggested by Gross (1998). The model describes five processes of ER that occur at different time points in the course of emotional processing and regulation. In this paper we focus on two ER strategies included in the process model of Gross—distraction and reappraisal—and also on labeling, an ER strategy that is not part of Gross' model. Reappraisal, distraction and labeling are cognitive strategies used to regulate emotions [for a review on the reciprocal relationship between emotion and cognition see Dolcos et al. (2011)]. We start with definitions and findings regarding distraction, reappraisal and labeling. Subsequently, we present the process model of Gross, with labeling as an additional form of ER. Finally, we suggest that labeling might mediate the effectiveness of reappraisal in clinical populations with deficits in emotion recognition and ER, and discuss the clinical implications of this suggestion and future directions of research and conceptualization.

## Distraction

Distraction is an antecedent-focused strategy of ER, meaning that it is implemented before the generation of the emotion. Distraction constitutes the deployment of attention away from a negative aspect of a situation, to a neutral or positive aspect (Gross, 1998). Attention can be deployed externally (e.g., focus on the shape of a certain stimulus) or internally (e.g., focus on neutral or positive thoughts). Distraction was found to be an effective ER strategy in various studies (Nolen-Hoeksema et al., 2008; McRae et al., 2009; Sheppes et al., 2011). Sheppes et al. (2011) found, for example, that people tend to choose distraction when emotional stimuli are highly intense, and it was also found that distraction reduces negative affect in depressed patients (Nolen-Hoeksema et al., 2008). Moreover, in cognitive behavioral therapy (CBT), patients learn how to distract themselves from negative situations that might cause dysphoria (Beck, 2011, p. 213). These findings are in line with data on brain activation during distraction that show increased activation in the ventro-medial prefrontal cortex (PFC) and decreased activation in the amygdala (McRae et al., 2009; Kanske et al., 2011).

## Reappraisal

Reappraisal is also an antecedent-focused strategy, but it is implemented later than distraction during the time course of ER (Gross, 1998). Reappraisal constitutes a cognitive change of the meaning of an emotion eliciting situation, in order to reduce negative feelings (Gross, 1998). Reappraisal was found to be highly adaptive and people who tend to use this strategy show greater well-being and fewer symptoms of depression compared with people who do not tend to use reappraisal (Gross and John, 2003). In addition, different studies demonstrated that when participants are explicitly asked to use reappraisal, they report less negative affect compared with a control group (e.g., Ochsner et al., 2004; Sheppes and Meiran, 2007). This is in line with studies that examined the neural basis of reappraisal and found increased activity in the medial, dorsolateral and ventrolateral PFC that was correlated with decreased activity in the amygdala while using reappraisal (Goldin et al., 2008; McRae et al., 2009). McRae et al. (2009) found that although distraction caused greater decrease in amygdala activation compared with reappraisal, reappraisal was more effective in down-regulating the emotional experience as measured by self-reports.

## Labeling

Labeling is the linguistic processing of the emotions that arise in a certain situation (Lieberman et al., 2007). Different studies showed that labeling, much like reappraisal and distraction, results in decreased activity in the amygdala and increased activity in prefrontal areas and Broca's area (Hariri et al., 2000; Lieberman et al., 2007; Torrisi et al., 2013; Tupak et al., 2014). Hariri et al. (2000) showed that labeling of facial emotions involved increased activation in the right ventral PFC and decreased activation in the amygdala compared with a control condition requiring matching facial stimuli with respect to emotional expressions. This finding suggests that labeling has a unique contribution to this pattern of brain activation since processing different characteristics of the emotional stimuli (e.g., by matching a facial expression) is insufficient to regulate amygdala activation. Similarly, Taylor et al. (2003) found that when participants rated their emotional experience while watching negative pictures there was decreased activation in the amygdala and increased activation in the dorsal medial PFC and the anterior cingulate sulcus. These findings regarding the pattern of activation in the brain during labeling were replicated in different studies (e.g., Nakamura et al., 1999; Narumoto et al., 2000; Gorno-Tempini et al., 2001) and suggest that linguistic processing of emotions (but not other, non-emotional properties of stimuli) regulates the activation in the amygdala. The influence of labeling on physiology was also demonstrated in a study by Tabibnia et al. (2008). In this study, spider phobics were assigned to three groups: exposure only, exposure with a negative label, and exposure with a neutral label. Eight days after the exposure to spider pictures, skin conductance response (SCR) was measured while watching the pictures from the initial exposure. Participants from the exposure with a negative label group showed lower SCR (indicating decreased physiological arousal) compared with the other two groups. In addition, their SCR was lower while watching new pictures of spiders compared with their SCR during their first exposure. In another study, spider phobics engaged in exposure to spiders while applying labeling, reappraisal, or distraction, or by watching the spiders (control group). It was found that when comparing the SCR immediately after exposure to that of 1 week after the exposure, the labeling group showed the greatest reduction in SCR compared with the other groups. On the other hand, the groups did not differ in their self-reported fear levels (Kircanski et al., 2012). It is important to distinguish between appraisal and labeling. Labeling is different from appraisal in the sense that appraisal is an automatic and general processing of various aspects of a situation [e.g., novelty, relevance; for review see Ellsworth and Scherer (2003); Brosch and Sander (2013)], and it includes a basic evaluation of emotional aspects in order to execute an adaptive emotional response (e.g., action tendencies; Brosch, 2013). Labeling, on the other hand, relates specifically to the emotional aspect of the situation and involves an explicit verbal process of identifying and naming the emotion (Lieberman et al., 2007).

## Time course of emotion regulation

The process model of ER (Gross, 1998) includes five processes of ER. The first process is situation selection—we choose whether to approach or avoid a situation. Next, there is situation modification—changing the situation. Then there is attentional deployment—changing the focus of attention (e.g., distraction). The fourth ER process is cognitive change (e.g., reappraisal)—changing the meaning of the situation. The last process is response modulation—control over the emotional response (i.e., behavioral, physiological, and experiential). In the time course of ER, consistent with Gross' model (1998), distraction starts to attenuate late positive potential (LPP) in the brain at early processes, 300 ms after the stimulus onset. Attenuation by reappraisal starts 1500 ms after the stimulus onset (Thiruchselvam et al., 2011), meaning that distraction reduces the emotional reactivity in the brain earlier than reappraisal. According to Gross' model, when a stimulus is presented we first appraise it (a process that starts 100 ms after the stimulus onset; see Brosch and Sander, 2013), and then implicitly decide whether to use distraction or reappraisal. However, it is important to note that ER is a continuous process, and different processes in the model can occur in parallel. We monitor our emotional response and can choose a preferable strategy to regulate emotions (consciously or unconsciously), and can switch between strategies. One can think about ER as a bottom-up or top-down process—it might be that Gross' model may describe a spontaneous or bottom-up process, but top-down control may enable switching strategies or changing the timing of strategy implementation.

## Our suggestion

**Figure 1:**
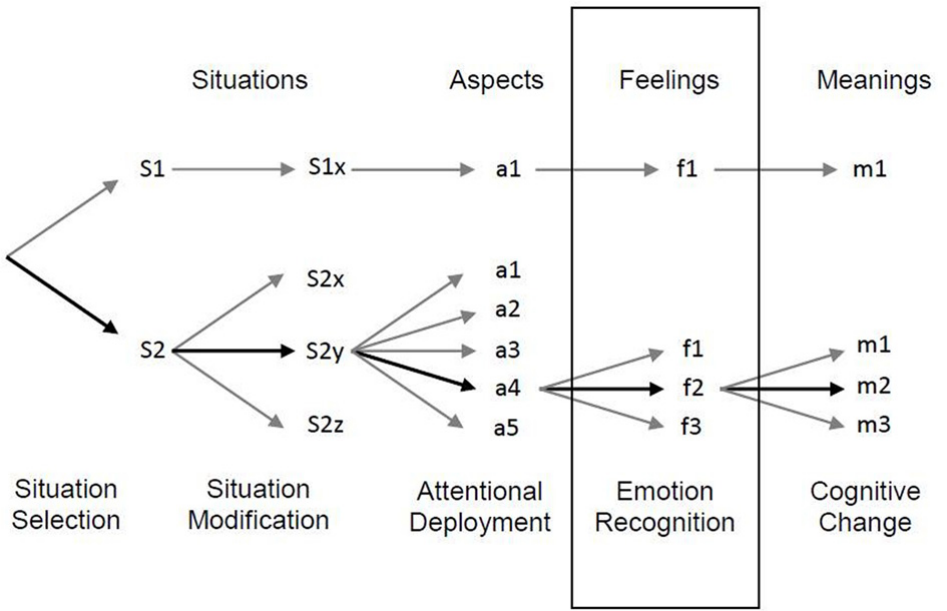
**A renewed model of the emotion regulation process, based on Gross' model (Gross, 1998; APA, adapted with permission)**. This model includes only antecedent-focused strategies and suggests that emotion recognition is part of emotion regulation processes.

We suggest that an additional stage of ER should be included in Gross' model (1998)—emotion recognition (e.g., labeling) (see Figure 1). Labeling itself is an ER strategy that helps to decrease emotional reactivity (Hariri et al., 2000; Lieberman et al., 2007; Tabibnia et al., 2008; Kircanski et al., 2012). It might be that similar to distraction, labeling allows dealing with highly intense emotional situations (e.g., exposure to phobic stimuli; Tabibnia et al., 2008; Kircanski et al., 2012), but unlike distraction, it also allows learning, since the individual attends to the emotional stimulus. Successful reappraisal includes an underlying process of emotion recognition (that is part of the appraisal process). Emotion recognition can be explicit (e.g., labeling) or implicit (e.g., awareness of the feeling). We suggest that healthy individuals succeed in reappraisal because they are able to recognize their emotions. This assertion is based on findings with subjects with alexithymia (difficulty to identify emotions and describe them; Aleman, 2005) who show a reduced tendency to use reappraisal compared with participants who do not suffer from alexithymia (Swart et al., 2009). Other studies also found a relationship between difficulties in emotion recognition and ER in different disorders (e.g., anorexia nervosa, Harrison et al., 2009; bipolar disorder, Getz et al., 2003). We suggest that when reappraising, we focus on the emotional situation and automatically (and maybe even unconsciously) appraise the situation, and recognize the feeling that arises from this situation and the action tendencies that evoked it. Only then we can interpret the situation differently. Different emotions elicit different action tendencies (e.g., fear elicits the need to move away from the danger), and in order to reappraise successfully, one has to modify this action tendency. In order to adaptively reappraise a situation and down-regulate a negative emotion, one has to attend to one's feelings and understand them, and not only appraise the situation in a general way. It might be that populations with difficulties in reappraising situations can benefit from explicit labeling of the emotions that a situation is eliciting, and labeling can be used as a mediator for successful reappraisal. Whereas distraction impairs memory of the emotional situation (Sheppes and Meiran, 2008), labeling enables focusing on the emotional situation. Therefore, we suggest that labeling allows coding the characteristics of the emotional situation and remembering the emotional experience, while reducing its intensity. Because the intensity of the emotional situation is lower after labeling (Tabibnia et al., 2008; Kircanski et al., 2012), it is possible to use reappraisal to further regulate the emotions elicited by the situation. For example, an individual with spider phobia (arachnophobia) can down-regulate his/her feelings during exposure when saying to him/herself “there are spiders here; I'm afraid, but they are little and harmless.” This sentence contains appraisal of the situation, labeling of the feelings and reappraisal of the situation that reduces the negative feeling. This example is in line with the use of labeling and the acknowledgement of its importance in cognitive therapy. In cognitive therapy, which can be viewed as a form of reappraisal training, identifying and labeling emotions is considered a cardinal therapeutic process, since it helps in understanding thoughts, beliefs and behaviors (Beck, 2011, p. 17). Moreover, patients who have difficulty identifying emotions are systematically taught to identify and label emotions, in order to improve their reappraisal capability (Beck, 2011, p. 94). The use of labeling was also suggested as one of the techniques that might help improve the outcomes of CBT for patients who suffer from obsessive-compulsive disorder (Abramowitz and Arch, 2013). We suggest that when studying ER processes in normal populations, emotion recognition occurs automatically and improves the ability to successfully reappraise; and therefore, the importance of explicit labeling was not evaluated properly.

## Future directions

Future studies regarding the effectiveness of reappraisal in clinical populations should consider the role of labeling in ER processes. It might be interesting, for example, to study if labeling followed by reappraisal is more effective than each strategy alone in clinical populations, and even in non-clinical populations. It is also important to understand what exactly should be labeled (e.g., the emotion itself, the antecedent that caused the emotion, etc.). To conclude, although the importance of emotion recognition, and specifically labeling, is common knowledge in psychotherapy practice, we suggest that emotion recognition should also be included in the theoretical models of ER. Future studies should investigate the cognitive costs of labeling and the relationship between labeling and reappraisal; specifically, the possibility that labeling has a crucial role in successful reappraisal in clinical populations.
